# Women’s experiences of physiotherapy for pregnancy-related pelvic girdle pain in primary care: a qualitative study at four months postpartum

**DOI:** 10.1136/bmjopen-2026-125092

**Published:** 2026-10-05

**Authors:** Annika Svahn Ekdahl, Annelie Gutke, Kaisa Mannerkorpi, Monika Fagevik Olsén

**Affiliations:** 1 Department of Health and Rehabilitation, University of Gothenburg Institute of Neuroscience and Physiology, Gothenburg, Sweden; 2 Department of Physiotherapy, Sahlgrenska University Hospital, Gothenburg, Sweden

**Keywords:** Pregnancy, QUALITATIVE RESEARCH, Pregnant Women, Person-Centered Care

## Abstract

**Abstract:**

**Objectives:**

To explore women’s lived experiences of physiotherapy for pregnancy-related pelvic girdle pain (PPGP), including how the care received corresponded to their needs and expectations.

**Design:**

Qualitative study using an inductive phenomenological approach. Individual semi-structured interviews were analysed using Giorgi’s Descriptive Phenomenological Psychological Method.

**Setting:**

Primary care physiotherapy clinic in Gothenburg, Sweden.

**Participants:**

10 women who had received physiotherapy for clinically verified PPGP were interviewed between March 2022 and September 2023. Participants were recruited from a primary care physiotherapy clinic and interviews were conducted by telephone or videoconference.

**Results:**

Women’s experiences of physiotherapy extended beyond symptom management and encompassed validation, reassurance and support in navigating the healthcare system. Three interrelated themes were identified: *On the threshold to physiotherapy*, describing how pain, reduced functioning, uncertainty about symptom legitimacy and social support influenced care-seeking; *A positive and reassuring encounter*, highlighting the importance of being listened to, receiving individualised advice and meeting a physiotherapist with expertise in PPGP; and *Need for proactive and collaborative maternity care*, emphasising the value of early information, timely access to physiotherapy, improved collaboration between midwives and physiotherapists and more flexible care pathways. Even when symptom relief was limited, participants considered individualised guidance, reassurance and validation meaningful outcomes of care.

**Conclusions:**

Women experienced tailored physiotherapy for PPGP as meaningful and reassuring. Their experiences of seeking physiotherapy were shaped by access to information, social support and guidance within the maternity healthcare system. Early information about PPGP, timely access to physiotherapy and continuity of care was described as important aspects of care, suggesting that these areas could be considered when developing care for women with PPGP.

**STRENGTHS AND LIMITATIONS OF THIS STUDY**

- Participants had clinically verified pregnancy-related pelvic girdle pain and received tailored physiotherapy based on current clinical guidelines in routine primary care practice.
- The phenomenological design enabled an in-depth exploration of women’s lived experiences and the meaning they attributed to physiotherapy.
- The sample was relatively homogeneous, with most participants being highly educated and born in Sweden or another European country, which may limit transferability to more diverse populations.

## Introduction

 Pregnancy can be a period of anticipation and joy; however, approximately 50% of pregnant women report experiencing pregnancy-related pelvic girdle pain (PPGP), a condition that can negatively affect daily life and well-being. Prevalence estimates vary widely depending on whether studies assess pelvic girdle pain specifically or include broader lumbopelvic pain, whether PPGP is clinically verified and the study design.^1–5^ PPGP may reduce work ability, limit physical activity, restrict participation in social activities and impair the capacity to care for older children.^3^ These limitations may lead women with PPGP to seek support from healthcare services.

For most women, PPGP resolves within a few months after delivery.^6^ However, approximately 16% of women who experienced pelvic girdle pain in pregnancy have been reported to have persistent PPGP at 3–6 months postpartum.^7^ For some women, symptoms may persist for as long as 12 years postpartum.^8^ The aetiology of PPGP is not yet fully understood, and several theories have been proposed, including muscular, hormonal, neurophysiological and inflammatory mechanisms.^9 10^ Symptoms of PPGP can develop at any time during pregnancy, most commonly around gestational week 18 or within 3 weeks postpartum. The pain is typically localised between the iliac crest and the gluteal folds and/or over the pubic bone and may present uni- or bilaterally, with or without radiation into the thighs.^1 2^

Almost all Swedish women are enrolled in the national maternity healthcare programme, which is free of charge, making the midwife their natural first point of contact for all pregnancy-related concerns. Previous research indicates that many women first consult their midwife for advice regarding PPGP, as they may be unaware about physiotherapy as a possible treatment option and/or where to seek physiotherapy.^11^ In Sweden, individuals can seek physiotherapy without a referral and may freely choose any clinic and as the healthcare system is tax-funded, patients pay a nominal fee. As of June 2026, 42 Swedish physiotherapists were formally recognised as specialists in obstetrics, gynaecology and urology. However, PPGP is also managed by physiotherapists working within musculoskeletal practice and by physiotherapists with an interest or further training in women’s health without formal specialist certification. Physiotherapy is recommended as a primary treatment for PPGP, and common interventions include exercises aimed at strengthening the trunk and hip muscles, the use of a pelvic belt and modalities such as acupuncture or transcutaneous electrical nerve stimulation.^12–14^

Previous research has highlighted the considerable impact of PPGP on women’s everyday lives and several challenges in their encounters with healthcare. Women have described limitations in daily activities, work and family life, as well as effects on emotional well-being and identity.^15–17^ They have also reported experiences of their symptoms being trivialised or not taken seriously, while valuing recognition of their condition, appropriate information and support from healthcare professionals.^15 16^ Women seeking physiotherapy for PPGP have described expectations of receiving an explanation for their symptoms, individually tailored advice and treatment, and support from a physiotherapist with expertise in PPGP.^11^ Postpartum, women with persistent PPGP have reported insufficient information and uncertainty regarding their symptoms and where to seek further support.^18^ More recent research in primary care suggests that satisfaction with physiotherapy may coexist with unmet needs, particularly regarding physiotherapists’ competence and the interventions provided.^19^

While previous qualitative research has provided important insights into women’s experiences of living with PPGP and their expectations when seeking physiotherapy, less is known about how women experience the physiotherapy they subsequently receive and whether it corresponds to their needs and expectations. Exploring women’s experiences after receiving physiotherapy may therefore provide further insight into which aspects of care are perceived as meaningful and where needs remain unmet. This study aimed to explore women’s experiences of physiotherapy for PPGP including how the care received corresponded to their needs and expectations.

## Method

### Design

Qualitative study with an inductive, phenomenological perspective. All women received oral and written information about the study and provided informed, written consent.

The study is reported according to the Standards for Reporting Qualitative Research.^20^

### Settings and participants

Eligible participants were women who had completed physiotherapy for PPGP in pregnancy at a primary care physiotherapy clinic in Gothenburg before being invited to participate and had sufficient proficiency in Swedish to provide written informed consent and participate in the interview. Exclusion criteria were any musculoskeletal/systemic disease affecting the lower back or pelvic region. No compensation or reimbursement was offered for participation.

An invitation to participate in the interview study was initially distributed to 16 eligible women 4 months postpartum as an attachment to a questionnaire for a cross-sectional study investigating the prevalence of PPGP 4 months postpartum.^21^ One woman recruited through the cross-sectional study accepted to participate. As recruitment for the interview study progressed slowly, recruitment continued independently after recruitment to the cross-sectional study had ended. Invitations were subsequently sent to 40 additional eligible women who had received physiotherapy for PPGP during their latest pregnancy, of whom nine participated. Thus, a total of 56 eligible women were invited and 10 participated. Eligible women received an initial invitation by mail, followed by one reminder and a final reminder by SMS and mail if they had not responded. Women who did not respond following the final reminder were considered non-responders. The participants could choose, according to their convenience, whether the interview would be conducted by telephone or via a digital meeting platform (Zoom).

The initial physiotherapy visit included a comprehensive history and systematic clinical examination to verify PPGP and differentiate it from lumbar pain.^22^ To be classified as PPGP, the pain was required to have its onset during pregnancy, be located between the posterior iliac crest and gluteal fold and/or over the pubic symphysis, with or without radiation into the thigh, and be reproducible by two or more pelvic pain provocation tests.^22^ Interventions followed current guidelines^1 12 13^ and were adapted to individual needs through clinical reasoning and shared decision-making. All participants received tailored advice on activity management, and most were offered a pelvic belt, pelvic floor muscle training and general trunk and hip exercises. At least one follow-up visit was scheduled, with additional sessions offered according to individual needs, treatment response and time remaining until delivery. A detailed description of the assessment and physiotherapy interventions has been reported elsewhere.^21^

The included participants (table 1) had a mean of 32.6 years, five were primipara and all were married/cohabitants. All were on maternity leave at the time of the interviews; they all had paid employment with various degrees of physical workload.

**Table 1:** Participant characteristics

Variable	Result n=10
Age, mean (range)	32.6 (26–38)
Country of birth, n	
Sweden	7
Other European country	3
Highest education, n	
High school	3
University	7
Parity, n	
1	5
2	3
≥3	2
PPGP debut, n	
Trimester 1	2
Trimester 2	8
PPGP in a previous pregnancy, yes	2 n=5

PPGP, pregnancy-related pelvic girdle pain.

### Researchers

The research team consisted of female physiotherapists with personal experience of pregnancy and experience in qualitative research (ASE, MFO, AG, KM) and clinical experience in treating women with PPGP (ASE, MFO, AG) and persistent pain (KM). One author (AG), a specialist in obstetrics, gynaecology and urology, was the physiotherapist treating all participants. To minimise the risk of bias related to this prior therapeutic relationship, she did not participate in the initial stage of the analysis.

### Procedure

10 individual interviews were performed between March 2022 and September 2023. To protect participants’ identities, each participant was assigned a numerical code (1–10), which was used throughout data analysis and when presenting quotations

The research team developed an interview guide with guiding questions covering topic areas related to the participants’ experiences (online supplemental file 1). The guide was pilot tested in one interview, which was not included in the analysis. Following the pilot interview, the opening question was revised from ‘receiving physiotherapy’ to ‘seeing a physiotherapist’ to encourage descriptions of the broader experience of the physiotherapy encounter rather than focusing solely on treatment received. The interviews explored reasons for seeking care, experiences of the physiotherapy encounter and interventions received, fulfilment of needs and overall satisfaction, as well as reflections on care for PPGP. Prompts and follow-up questions were used when needed to encourage rich and detailed descriptions of the women’s experiences.

The interviews were performed by the first author (ASE); audio recorded and began with the initial question:

I am interested in hearing about your experience of seeing a physiotherapist for the pelvic girdle pain you had during your most recent pregnancy. Could you tell me about your situation when you decided to seek healthcare for your symptoms?

One interview was conducted via Zoom, the other nine by telephone. Interview duration varied between 18 and 35 min. The duration of the interviews reflected individual differences in how participants described their experiences and were not considered a measure of phenomenological depth. The interviewer used probing and follow-up questions when needed to facilitate concrete and detailed descriptions of lived experiences. All interviews provided sufficiently rich experiential descriptions to contribute to the phenomenological analysis of the phenomenon under study: women’s experiences of seeing a physiotherapist for pelvic girdle pain during their most recent pregnancy.

The interviews addressed experiences and perceptions of pain, which could potentially evoke emotional discomfort. Participants were informed that they could withdraw from the study at any time without providing a reason. Although no direct clinical benefit was expected, participation offered an opportunity to share personal experiences and contribute to knowledge that may inform future treatment and preventive strategies for PPGP. Based on previous experience of the research team, women with PPGP often wish to talk about their experiences but are seldom given the opportunity to do so.

### Analysis

The interviews were conducted by the first author (ASE) and analysed according to the Descriptive Phenomenological Psychological Method developed by Giorgi.^23^ This method seeks to describe lived experiences, their meanings, and underlying structures.^24^

Data collection and analysis were performed within the framework of *phenomenological reduction*, meaning that the researchers’ prior knowledge and preconceptions about the studied phenomenon were set aside, or bracketed, to approach the data with openness.^23^ This bracketing process aimed to ensure that the phenomenon, that is, the women’s experiences of physiotherapy for PPGP, was described as it presented itself, without assumptions. Reflection and discussion among the authors throughout the analytic process supported this attitude of openness.

All recorded interviews were transcribed verbatim. The analysis began with repeated readings of each transcript to gain a sense of the whole description. ASE then identified meaning units, that is, text segments expressing aspects of the participants’ experiences of the phenomenon. These meaning units were subsequently discussed with MFO and AG and transformed into expressions of their psychological meaning in relation to the phenomenon under study. All authors participated in the final stage of the analysis, where the transformed meaning units were synthesised, repeatedly read, discussed and revised until the essential structure of the studied phenomenon could be described. The structure of the phenomenon is understood as an interplay between its constituents, where each one is essential and the removal of any constituent would alter the structure itself.^23^

### Patient and public involvement

No patients or members of the public were involved in design, conduction or interpretation of the study.

## Results

The interviews gave rich descriptions of the women’s lived experience of physiotherapy for PPGP. The analysis revealed that women’s experiences encompassed the process of seeking care, being met and validated in the clinical encounter and reflecting on how maternity healthcare pathways could be improved. Social support influenced both care-seeking and adherence, while the physiotherapy consultation was generally experienced as thorough, reassuring and professionally grounded. At the same time, the women identified structural aspects of care, such as access, information and collaboration between healthcare providers, as important areas for improvement.

The experience of physiotherapy for PPGP is presented as three themes, each composed of constituents that together illuminate the essential meaning of the phenomenon (table 2). Quotations are presented selectively in the text to illustrate the analytic interpretation, consistent with the aim of describing the essential structure of the phenomenon rather than reproducing individual narratives.^23^

**Table 2:** Experiences of physiotherapy for PPGP presented as three themes and their constituents

On the threshold to physiotherapy	A positive and reassuring encounter	Need for proactive and collaborative maternity care
Doubting the symptoms’ legitimacy and struggles of acceptance	Being seen and taken seriously	Early information reduces concern and facilitates care-seeking
Limited functioning and running out of strategies	Empowerment through tailored advice	Importance of timely and early access
Expectations shaped by personal and others’ experiences	Unexpected thoroughness and advice that reshaped assumptions	Increase collaboration between midwife and physiotherapist
Social support as a facilitator of care		Barriers to accessible and flexible care

PPGP, pregnancy-related pelvic girdle pain.

### On the threshold to physiotherapy

The act of seeking care for PPGP began as a response to pain and limitations in daily functioning. For some, this step was accompanied by doubt about the legitimacy of their symptoms and feelings of guilt related to reduced functioning. Social support from partners, family, colleagues or friends emerged as an important factor that validated their experiences, facilitated care-seeking and compliance with interventions.

#### Doubting the symptoms’ legitimacy and struggles of acceptance

The women described the beginning of their care‐seeking as shaped by doubt, hesitation and a struggle to accept the impact of their pain. Many expressed uncertainties about the legitimacy of their symptoms and tended to downplay the significance of their limitations, questioning whether their complaints are ‘serious enough’ to justify seeking help. Alongside this, feelings of guilt and shame emerged; guilt about not being able to work or needing sick leave, guilt about requesting help with pain management, and guilt about not having exercised enough before pregnancy.

I think a lot of women probably have these problems. But you tell yourself that it’s normal—or however you should put it—to be in pain. That you're supposed to be in pain during pregnancy, or that it’s your own fault because you didn't exercise enough, or whatever the reason might be. And then you… I know other women who just carry on and put up with it instead of seeking help. (Participant 10)

Pregnancy itself, particularly a first pregnancy, was described as a period involving substantial adjustments that may cause concern. Some women reflected on societal norms and expectations about how a pregnancy or a pregnant woman should be, and also the pressure to return to ‘pre-pregnancy state’ quickly postpartum. These external expectations contrasted with their own experiences and contributed to feelings of concern and self‐doubt.

There’s also a lot of pressure from outside to bounce back, and you’re supposed to exercise, and you’re supposed to get strong again, and have your best ‘mum body’, and be able to carry your children, sort of. (Participant 10)

A strong desire to remain independent in daily life appeared centrally; they hoped for advice that could enable them to continue working and maintaining their usual levels of physical activity. For women navigating multiple pregnancy-related complaints, the overall burden increased. Some women described that postpartum the focus shifted entirely to the baby’s well-being, leaving little room for attention to their own recovery.

#### Limited functioning and running out of strategies

Women described turning to physiotherapy when pain and functional limitations disrupted daily life and when their own strategies for managing pain had been exhausted. Pain undermined their perceived capacity and affected both professional and personal roles. They experienced limitations at work, at home, and in their physical activity, and these restrictions could negatively influence their mental well-being.

But this time it was much worse than the first. Yeah, I couldn’t … I had a hard time working because I was in so much pain, even more afterwards. And at home I could hardly … It was difficult to play with my oldest son, and it was difficult to sit on the sofa. And it was … everything became difficult … And I had a really hard time sleeping because I couldn’t lie on my sides. (Participant 7)

Acute worsening of pain generates concern and insecurity, often leading to reduced physical activity and thereby further in their daily functioning. Workload demands and everyday tasks were described as provoking pain and intensifying its consequences. Many women were also concerned that their pain would worsen as the pregnancy progressed or that it might persist after delivery. The ability to remain physically active and manage daily life was described as essential to maintaining well-being, making the constraining effects of PPGP especially burdensome.

#### Expectations shaped by personal and others’ experiences

Past experiences, both the participants’ own and those shared by others, shaped expectations about physiotherapy. For women who had experienced PPGP in a previous pregnancy, that familiarity could reduce concern and made the current situation feel more manageable. Prior exercise experience also contributed to a sense of autonomy and control. Personal knowledge of PPGP and awareness of physiotherapy as an option for help appeared to ease concern and facilitate care-seeking. Women described receiving a mixture of reassuring and discouraging information from people close to them. These shared experiences influenced how they anticipated physiotherapy might help.

And my mother has had pelvic girdle pain, and I have a friend who was also pregnant and had really bad problems with pelvic girdle pain. So they were both really insistent: ‘Get help straight away, because if you get help early, it can stop it from getting worse.’(Participant 1)

#### Social support as a facilitator of care

Encouragement from partners, family, friends or colleagues helped women validate their symptoms and reduced the threshold for seeking physiotherapy. Practical support, such as assistance with childcare, facilitated attendance. Conversely, lack of support could make access to care more difficult, leading some women to refrain from seeking help, choosing instead to endure the pain and hope it improves after the delivery. Social support was also important for adherence to interventions; encouragement helped women complete exercises despite fatigue. Group training was described as another form of social support for motivating those who struggled to exercise alone.

It felt difficult. That’s why it took me such a long time to do something about it, because I kept thinking, 'Well… it’s not that bad today. I'm fine. I'm just exaggerating it. It’s really nothing, not like that…' But in the end, my husband told me that I actually had to do something about it because it wasn't sustainable for me to keep putting up with that much pain. (Participant 7)

### A positive and reassuring encounter

Meeting the physiotherapist was described as a positive and reassuring encounter. The women felt seen and taken seriously; their complaints were not trivialised. A thorough examination, the physiotherapist’s expert knowledge and a pedagogical and professional approach created a sense of security and trust. Tailored advice for managing pain in everyday life was experienced as useful, even when the effects were not always as expected.

#### Being seen and taken seriously

The women described the encounter with the physiotherapist as a positive and reassuring experience. Being seen, listened to, and taken seriously was highlighted as essential. They valued meeting a physiotherapist who demonstrated knowledge about PPGP and who showed interest in how they managed their everyday life.

I think it’s also important to have someone who is particularly interested in helping women in this situation. And you know, there’s an important psychological aspect to it as well - feeling that you’re seen. (Participant 3)

Even when pain relief or symptom improvement did not fully meet their expectations, the physiotherapy consultation was still perceived as valuable. The sense of being acknowledged and having their complaints validated appeared to be a central aspect of the positive experience.

#### Empowerment through tailored advice

The participants expressed satisfaction with the physiotherapy they received, emphasising the importance of having interventions tailored to their individual needs. Expertise combined with a professional and pedagogical approach from the physiotherapist increased their confidence regarding pain management and reduced concern about the symptoms. The women appreciated receiving answers to their questions and gaining guidance that made sense in relation to their specific life situation and facilitated pain management. The tailored advice that took their unique circumstances and activities into account was described as highly positive and the advice was described as useful even after pregnancy. Several types of interventions were described, including advice on managing everyday tasks and recommendations for finding less painful ways to remain physically active.

Yes, she listened to me and took all my thoughts and questions into account. When I told her I wanted to continue working rather than just stay at home, it felt as though we shared the same goal. It became her goal too - that I should be able to keep working instead of just staying at home. So… yes, I felt a sense of partnership with her. (Participant 6)

#### Unexpected thoroughness and advice that reshaped assumptions

Several women were surprised by the thoroughness of the initial consultation. They did not expect the visit to last around 60 min, nor did they anticipate receiving such a detailed examination and explanation of PPGP and possible interventions. Compared with prior experiences of healthcare, for most participants, the consultation exceeded their expectations.

I don't think I expected to be treated this well beforehand. But afterwards, I felt that it was probably the most important thing. Yes, I was positively surprised, if you can say that. I thought I received really good care, and the assessment was very thorough. (Participant 8)

Some women described how they also were surprised by certain types of advice, for instance, instructions to limit physical load or avoid stairs. They had assumed that moving as much as possible would be beneficial and did not expect recommendations to reduce certain activities.

### Need for proactive and collaborative maternity care

Suggestions for improving care emerged. The women described the value of closer collaboration between maternity healthcare and physiotherapy, emphasising the importance of timely access and early information about possible pregnancy-related symptoms and the availability and access to physiotherapy both in pregnancy and postpartum.

#### Early information reduces concern and facilitates care-seeking

The women highlighted a need for early information about possible pregnancy-related complaints, including PPGP, to help them feel prepared. Many expressed that if they had known more about PPGP and where to seek care, they would likely have contacted a physiotherapist earlier. They wanted to know early in pregnancy that physiotherapy was an available option for promoting well-being and managing emerging symptoms. Early information was therefore described to reduce uncertainty and encourage timely care-seeking.

I think physiotherapy for pelvic girdle pain could be promoted more. I think a lot of women probably suffer unnecessarily when there are fairly simple things that can help. My midwife was actually aware of it and did mention it, but I think it could be brought up even earlier in pregnancy and presented more as something preventive - what you can do to reduce the risk or manage the symptoms. It could even be part of the standard programme of antenatal care. (Participant 2)

#### Importance of timely and early access

The women emphasised the importance of timely access to physiotherapy. They described early intervention as a preventive factor, noting that delays of even a few weeks may lead to increased pain as pregnancy progressed. Early access was also seen as central for maintaining a sense of control and preventing unnecessary concern. Overall, they expressed a strong desire for an efficient care process where physiotherapy clinics could be reached without delay.

No, but I also think that when you’re pregnant, it’s important to be seen fairly quickly, so you don’t have to wait too long. It often gets quite a lot worse quite quickly because you get heavier and so on. So I suppose it’s about feeling that you get help in time, maybe… and getting a quick answer that it isn’t anything dangerous. You’re a bit worried when you’re pregnant. (Participant 8)

#### Increase collaboration between midwife and physiotherapist

The midwife was described as the women’s natural first contact, yet they reported uncertainty about what information midwives provided regarding PPGP. Several women noted a significant time gap between the first registration visit at the maternity healthcare centre (gestational weeks 4–12) and the next appointment around gestational week 25. During the period between the visits, they were unsure whether they should have contacted the midwife regarding developing symptoms.

Several women described that midwives identified their need for treatment and encouraged them to seek physiotherapy, but they felt that direct referral to a specific clinic, or to a physiotherapist working within the maternity healthcare unit, would have made the process more straightforward. They expressed that better collaboration and clearer routines between midwives and physiotherapists, especially regarding early information and referral pathways, could improve care for women with PPGP.

I think it’s really good if it [pelvic girdle pain] is picked up early at the maternity clinic. It gives you a point of contact, and you also get information there, and they let you know early on that, ‘if you need to, then …’ Yeah, something like that. I think … it was good. My midwife immediately said to me, ‘I think you should call the physiotherapist.’ (Participant 3)

#### Barriers to accessible and flexible care

Women described logistical barriers that made in-person visits challenging. Travelling to the clinic could worsen PPGP, and some reported increased pain for the remainder of the day after appointments. They therefore requested more flexible alternatives, such as digital or telephone follow-ups, particularly when adjustments to treatment were needed.

It would have been really helpful [to have follow-up appointments by phone or online], because at that time only the first appointment could be done digitally. After that, I just had to get on my bike and cycle to the clinic because walking was too painful. By the time I got home, the whole day was basically gone. The pain was so much worse afterwards that I couldn't do anything else that day. (Participant 9)

Some experienced the care process as slow and inflexible, feeling required to trial one intervention at a time with mandatory in-person follow-ups before changes could be made. This was frustrating, especially when they felt insufficiently informed about available options.

Women unfamiliar with physiotherapy described difficulties locating appropriate services, particularly clinics with expertise in treating pregnant women. Without referral from a midwife or informal guidance from friends or family, it was often unclear where to seek care.

## Discussion

The findings suggest that women’s experiences of physiotherapy for PPGP extend beyond symptom management to include validation, reassurance and navigation of the healthcare system. While the physiotherapy encounter was generally perceived positively, access to timely information, social support and collaborative maternity care emerged as central to facilitating care-seeking and promoting well-being.

The pathway to seeking physiotherapy for PPGP can be experienced as either straightforward or challenging, depending on access to information, social support and guidance from healthcare professionals. When women knew where to seek care or were actively referred by a midwife to a physiotherapist with expertise in PPGP, access required little effort. In contrast, those without prior knowledge or guidance from midwives, family, friends or colleagues described care-seeking as demanding and exhausting. This is particularly relevant given that pain and reduced functioning already place a substantial burden on everyday life. Similar experiences of uncertainty about where to turn for help have been described among individuals with persistent pain,^25^ suggesting that difficulties in navigating healthcare systems may be a broader issue for people living with pain.

Accepting pain and reduced functioning emerged as a significant challenge, as the women valued independence and the ability to engage in meaningful activities essential for both physical and mental well-being. Being able to function and manage everyday life independently has been described as a fundamental aspect of health.^26 27^ In this study, seeking physiotherapy was motivated not only by pain relief but also by a desire for strategies to manage everyday activities and not having to rely on others for help. Doubts about the legitimacy of PPGP and whether care-seeking was justified were common and may act as barriers to accessing care. Such doubts are consistent with previous research describing experiences of mistrust or minimisation of symptoms within healthcare encounters,^28^ highlighting the vulnerability of women with this pregnancy-related pain.

Meeting a physiotherapist with specific knowledge of PPGP, a professional attitude and a pedagogical approach was described as central to a positive care experience. Feeling seen, listened to and receiving individualised advice tailored to one’s unique life situation contributed to reassurance and empowerment as the women described how they doubted symptoms’ legitimacy and had run out of strategies for self-care. These findings align with previous research demonstrating that women value expertise and individualised interventions when seeking physiotherapy for PPGP^11 16^ and are consistent with expectations expressed prior to treatment.^11^ Even if symptom relief was limited, validation and understanding were perceived as meaningful outcomes of care. Reassurance and validation of symptoms and the pedagogical approach from the physiotherapist reduced pain-related concern and facilitated pain management. Women can have different assumptions about PPGP, and physiotherapy consultations may facilitate an understanding of the complaints and provide reassurance through explanations based on the individual’s knowledge and concerns.^29 30^ This underscores the importance of validating patients’ perceptions, as such validation may influence healthcare-seeking behaviour, with both physiotherapists and midwives playing key roles.^29^

A recurring theme was the participants’ wish for earlier and clearer information about possible pregnancy-related symptoms, including PPGP. Early knowledge about what symptoms may arise and when and where to seek care was perceived to reduce concern and facilitate timely care-seeking. Pain education has been shown to reduce worry and increase autonomy and control, particularly when provided early in pregnancy.^31 32^ Furthermore, education combined with interventions such as exercise appears to be more effective than education alone.^32 33^ Exercise is recommended as a first-line intervention for PPGP,^12 13^ and integrating education and active treatment strategies may be a preferable treatment strategy for PPGP.

Postpartum, the women described how attention from both themselves and healthcare professionals shifted primarily toward the baby, leaving their own recovery and persistent pain concerns in the background. In Sweden, women are offered structured follow-ups to monitor both physical and mental recovery through maternity healthcare up to several weeks postpartum, after which the focus largely transitions to child health services. Consistent with earlier research, PPGP may therefore lose priority despite ongoing symptoms.^18 34^ A closer collaboration between physiotherapists and midwives could help maintain continuity of care, reduce concern and support recovery after delivery^35^ and this was emphasised by the participants.

Meeting a physiotherapist with expertise and interest in pregnancy-related complaints was perceived as an important factor for reducing concern and increasing empowerment through tailored advice. This finding is relevant in light of research reporting that women with PPGP attending primary care physiotherapy clinics perceived a lack of competence regarding PPGP^34^ and expressed a need for more physiotherapists specialised in women’s health.^19^

Barriers to attending physiotherapy, such as transport difficulties, limited information about the relevance of physiotherapy and passive treatment approaches, have also been identified as reasons for non-attendance.^36^ Alternative care models, including home visits, may increase satisfaction and accessibility, as shown in postpartum care contexts,^37^ and could be relevant for women with severe PPGP.

Previous research has described women’s expectations when seeking physiotherapy, including individualised advice and access to a physiotherapist with expertise in PPGP.^11^ Our findings add to this by highlighting that when these expectations were met, physiotherapy was experienced as meaningful and reassuring. They also suggest that women’s experiences were influenced by factors beyond the physiotherapy encounter itself, including information about PPGP, access to physiotherapy, guidance from midwives and continuity of care.

Our findings are in line with Eklund and Sjöström,^19^ who found that overall satisfaction with physiotherapy for PPGP could coexist with unmet needs regarding physiotherapists’ competence and the interventions provided. The present study extends this understanding by highlighting the importance women placed on reassurance, individually tailored support and PPGP-specific expertise, while also drawing attention to the role of information and guidance within maternity healthcare in their experiences of seeking physiotherapy.

### Strengths and limitations

Several aspects of the findings are consistent with previous qualitative studies conducted in other settings,^16 29 31 34^ suggesting that similar experiences may occur across different healthcare contexts. Another strength of this study is that all participants had clinically verified PPGP and were recruited from the ordinary clinical flow of a physiotherapy clinic located in a socioeconomically representative urban area in Sweden, supporting the credibility of the findings. A potential limitation is that one of the authors (AG) was the physiotherapist treating all participants, which may have influenced participants’ experiences of the encounter. However, this could also be a strength as all women received interventions based on current guidelines and tailored to their specific needs.

The aim of the phenomenological analysis was not generalisability but to provide a rich and nuanced description of lived experience of physiotherapy. Therefore, data saturation was not used to determine sample size, as saturation is not a methodological requirement in Giorgi’s descriptive phenomenological approach. The study included ten participants, which is appropriate for research that prioritises depth and detailed exploration of lived experience over breadth. The sample allowed for variation in experiences while maintaining analytic depth consistent with Giorgi’s method.^23 38^ Although the aim was to recruit a broad range of participants, most women had higher education, were born in Sweden or other European countries, and were married or cohabiting. We believe that this study contributes to a deeper understanding of women’s experiences of physiotherapy for PPGP, including how the care received corresponded to their needs and expectations. However, the participants were recruited from a single primary care physiotherapy clinic and constituted a relatively homogeneous group. Different healthcare settings or participant populations may reveal additional variations in how the phenomenon is experienced, which should be considered when assessing the transferability of the findings.

Participation was based on self-selection, and women who chose to participate may have had different experiences of physiotherapy than those who declined or did not respond. This may have influenced the range of experiences represented in the findings. In addition, no analysis was conducted of women who were non-responders, and their experiences may differ from those who chose to take part. As participants were interviewed 4 months postpartum about physiotherapy received during pregnancy, some recall bias cannot be excluded; however, the interviews focused on their experiences of care rather than recall of specific treatment details.

Several strategies were applied to enhance trustworthiness of the study. In-depth interviews provided rich descriptions of women’s lived experiences. All authors were women with personal experience of pregnancy, which may have provided familiarity with the context while also introducing pre-understandings that could influence the interpretation. Within the phenomenological approach, the researchers sought to restrain these pre-understandings and remain attentive to participants’ descriptions throughout the analysis. Ongoing discussions among the authors supported reflexivity and shared interpretation. Detailed descriptions of the context, participants and analytic procedures facilitate assessment of transferability. The data were repeatedly reviewed, and emerging structures were discussed and refined among the authors until consensus was reached.^39^

## Conclusion

Tailored physiotherapy for PPGP provided by an expert physiotherapist was experienced as meaningful and reassuring. Experiences of seeking physiotherapy were shaped not only by pain and functional limitations but also by access to information, social support and guidance within the maternity healthcare system. Women described early information about PPGP, timely access to physiotherapy and continuity of care across pregnancy and the postpartum period as important aspects of care. These findings suggest that collaboration between midwives and physiotherapists, together with flexible and accessible care pathways, could be considered when developing care for women with PPGP.

## Supplementary material

10.1136/bmjopen-2026-125092 online supplemental file 1

## Data Availability

Data's are available upon reasonable request.
