# Supplementary material for: Women’s experiences of physiotherapy for pregnancy-related pelvic girdle pain in primary care: a qualitative study at four months postpartum
Source: BMJ Open. 2026 Oct 5;16(10):e125092. doi: 10.1136/bmjopen-2026-125092 (PMC13646931; doi:10.1136/bmjopen-2026-125092)
Supplement: Supplementary file 1 [file bmjopen-2026-125092.reviewer_comments.pdf]

# PEER REVIEW HISTORY

BMJ Open publishes all reviews undertaken for accepted manuscripts. Reviewers are asked to complete a checklist review form and are provided with free text boxes to elaborate on their assessment. These free text comments are reproduced below.

## ARTICLE DETAILS

### Title (Provisional)

Women's experiences of physiotherapy for pregnancy-related pelvic girdle pain in primary care: a qualitative study at four months postpartum

### Authors

Svahn Ekdahl, Annika; Gutke, Annelie; Mannerkorpi, Kaisa; Fagevik Olsén, Monika

---

## VERSION 1 - REVIEW

---

| | |
|---|---|
| **Reviewer** | **1** |
| **Name** | **Mose, Ayenew** |
| **Affiliation** | **Monash University, Obstetrics and Gynaecology** |
| **Date** | **01-Aug-2026** |
| **COI** | **None** |

---

Thank you for the opportunity to review this interesting qualitative study exploring women's experiences of physiotherapy for PPGP. The topic is clinically relevant, and the manuscript is generally well written with a clear clinical focus. The use of a phenomenological approach is appropriate for exploring women's lived experiences. However, several methodological and reporting issues require clarification before the manuscript can be considered further.

Abstract

The conclusion appears to overstate the implications of the findings, considering the qualitative design and the small, relatively homogeneous sample (n=10). The authors can moderate their conclusions.

Introduction

The opening section would benefit from a stronger epidemiological context regarding the prevalence and clinical impact of PPGP.

The authors should clarify more explicitly how this study provides novel insights beyond existing qualitative literature on women's experiences of PPGP and physiotherapy interventions.

Methods

Interviews lasted between 18 and 35 minutes. The authors should provide further justification regarding how sufficient phenomenological depth was achieved, particularly for interviews at the shorter end of this range.

The recruitment process requires further clarification. Of the 56 eligible women invited, only 10 participated, representing an approximately 18% response rate. The authors should discuss the potential for selection bias and whether women who declined participation may have had different experiences or perspectives.

Please clarify the recruitment procedures: how many times were eligible participants contacted, and at what point were non-respondents considered as declining participation?

Did the authors offer any compensation or reimbursement for participants' time, and if not, please provide justification?

Results

It would strengthen the interpretability of the findings if brief participant characteristics (e.g., parity, pregnancy stage) were provided alongside participant quotations, rather than only identifying participants as Participant 1, Participant 2, etc. This information could be presented while maintaining anonymity.

Overall assessment

This manuscript addresses an important and clinically relevant topic and has the potential to make a valuable contribution to the literature. Addressing the methodological and reporting issues above would strengthen the interpretability of the findings.

---


**Reviewer** **2**

**Name** **Ceprnja, Dragana**

**Affiliation** **Western Sydney University, Department of Physiotherapy, Westmead Hospital**


**Date** **04-Aug-2026**

**COI** **None**

---

Thank you for this study which adds to the body of evidence relating to qualitative enquiry in PPGP. Please see my comments below.

Thank you for this study which adds to the body of evidence relating to qualitative enquiry in PPGP.

Introduction

Line 92 – suggest change language from "affected women" to women with PPGP

Line 94 – can you provide estimate of how many women experience persistent pain? Eg. %

Line 112 – do you have any data regarding how many physiotherapists in Sweden have interest or further training in Womens health, noting PPGP may also be managed by musculoskeletal physiotherapists considering it could come under MSK umbrella

Would be good to include a paragraph that summarises previous qualitative research with women with PPGP and what this has found to then establish the gap your study is filling.

Method

Line 127 – change word "got" for received, eg. All women *received* oral and written information ….

Line 131 – can you provide more detail about the timeframe that women received treatment for PPGP. Were they invited to participate after they had complete their physiotherapy sessions or while they were still receiving physiotherapy? This may introduce recall bias if after physiotherapy. How long after were they seen?

Line 144 – Provide more detail regarding how PPGp was verified. What assessments were completed?

Line 149 – Provide data regarding how many physiotherapy visits the participants had? Range, average, could add to Table 1 as well

How was the sample size determined? With qualitative studies this usually involves saturation of data. Can you please provide information regarding this? (links to SRQR checklist - Sampling strategy)

It looks like you assigned a code to participants for de-identification. Can you please add a line or two about this (links to SRQR checklist – Data processing)

Did you use an interview guide? How was this developed and piloted? (links to SRQR – Data collection instruments and technologies)

Did you undertake member checking? If so, please add in details. If not, why not? (links to SRQS – techniques to enhance trustworthiness)

Line 191 – add the following to the sentence, ie. the women's experiences *of physiotherapy for PPG,* was described as it was …..

Line 195 – the sentence is unfinished, to get a sense of the whole *range of topics discussed?*, or the whole *range of women's narratives*? Select one of these or something similar to finish off the sentence.

Results

This is well written.

I would suggest moving the quotes to the constituent sections where they would be more illustrative than at the introduction of the theme for all three themes.

Table 2 – I think the correct grammar would be "*Need* for proactive and collaborative maternity care rather than "Needs", same for Line 340 and in the Abstract section under results.

Discussion

Another strength is that your themes are similar to other studies that have been conducted elsewhere in the world, eg. refs 23, 24, 26, 30

Were all your authors women? This may be a limitation worth noting as well if so.

Would also be of benefit to include a few lines about the gap in evidence your research has filled, ie. there are clear links with previous studies here but what is unique about your study? What particular gap has been filled?

SRQR reporting checklist – could strengthen some of the checklist as already highlighted in comments above

---

## VERSION 1 - AUTHOR RESPONSE

**Reviewer: 1**
**Mr. Ayenew Mose, Monash University, Wolkite University**

**Abstract**
**The conclusion appears to overstate the implications of the findings, considering the qualitative design and the small, relatively homogeneous sample (n=10). The authors can moderate their conclusions.**

***Reply:*** *We agree that the implications of the findings should be interpreted with consideration of the qualitative design and the relatively small and homogeneous sample. We have therefore revised the conclusions in both the abstract and the main text to remain more closely grounded in participants' experiences and to avoid implying effects of specific changes to care that were not examined in this study.*

**Introduction**
**The opening section would benefit from a stronger epidemiological context regarding the prevalence and clinical impact of PPGP.**
***Reply:*** *We have strengthened the epidemiological context by clarifying that reported prevalence varies considerably across studies depending on the definition of PPGP, whether the condition is clinically verified, and study design. We have also added information on the prevalence of persistent PPGP postpartum and retained information on its impact on everyday functioning. P. 4 lines 90-98.*

**The authors should clarify more explicitly how this study provides novel insights beyond existing qualitative literature on women's experiences of PPGP and physiotherapy interventions.**

***Reply:*** *We have revised the Introduction (p. 5, lines 120-139) to more clearly position the present study in relation to previous qualitative research. Previous studies have explored women's experiences of living with PPGP and their expectations when seeking physiotherapy. We have clarified that the present study extends this literature by exploring women's experiences after receiving physiotherapy, with particular attention to how the care received corresponded to their needs and expectations. We have also incorporated recently published qualitative research to provide an updated context for the study.*

**Methods**
**Interviews lasted between 18 and 35 minutes. The authors should provide further**

**justification regarding how sufficient phenomenological depth was achieved, particularly for interviews at the shorter end of this range.**

***Reply:*** *We agree that sufficient phenomenological depth is essential. However, in descriptive phenomenological research, the duration of an interview is not in itself an indicator of the richness or depth of the description obtained. Participants varied in how extensively they verbalized their experiences, and a longer interview did not necessarily result in a richer description of the phenomenon. During the interviews, follow-up questions were used to encourage participants to elaborate on concrete experiences, thoughts, feelings, and situations relevant to the phenomenon. The shorter interviews were considered adequate because they provided sufficiently rich and concrete descriptions of the participants' lived experiences to enable identification and transformation of meaning units and to contribute to the description of the phenomenon's general structure. We have clarified this in the manuscript on p. 8, lines 213-219.*

**The recruitment process requires further clarification. Of the 56 eligible women invited, only 10 participated, representing an approximately 18% response rate. The authors should discuss the potential for selection bias and whether women who declined participation may have had different experiences or perspectives.**
**Please clarify the recruitment procedures: how many times were eligible participants contacted, and at what point were non-respondents considered as declining participation?**

***Reply:*** *We have clarified the recruitment procedure in the Methods section, p. 5-6, lines 148-163. Initially, 16 eligible women were invited to the interview study through a concurrent cross-sectional study, of whom one participated. As recruitment to the interview study progressed more slowly than anticipated, recruitment continued independently after recruitment to the cross-sectional study had ended. An additional 40 eligible women who had received physiotherapy for PPGP were invited, of whom nine participated. Eligible women received an initial invitation by mail, followed by one postal reminder and a final reminder by SMS and mail. Those who did not respond after the final reminder were considered non-responders.*
*We agree that self-selection may have influenced the experiences represented and have added this to the limitations in p. 20, lines 574-578.*

**Did the authors offer any compensation or reimbursement for participants' time, and if not, please provide justification?**
***Reply:*** *No financial reimbursement or other compensation was offered to the participants. This information is added to p. 6, line 152. In Sweden, participant compensation is not routinely provided in research studies, and participation without compensation is not uncommon.*

***Results***
**It would strengthen the interpretability of the findings if brief participant characteristics (e.g., parity, pregnancy stage) were provided alongside participant quotations, rather than only identifying participants as Participant 1, Participant 2, etc. This information could be presented while maintaining anonymity.**

***Reply:*** *Thank you for this suggestion. We considered this approach but chose to present participant characteristics in Table 1 rather than alongside individual quotations. Quotations are used selectively to illustrate the analytic interpretation rather than to represent individual*

*participant narratives, consistent with the phenomenological aim of describing the essential structure of the phenomenon across participants. We have therefore retained the current presentation.*

-------------------------------------------------------------------------------------------------------------------
-

**Reviewer: 2**
**Dr.  Dragana Ceprnja, Western Sydney University**

**Introduction**

**Line 92 – suggest change language from "affected women" to women with PPGP**
***Reply:*** *We agree, the wording has been changed accordingly on p. 4, line 94*

**Line 94 – can you provide estimate of how many women experience persistent pain? Eg. %**
***Reply:*** *Yes, we have added this information on p. 4, lines 96-98*

**Line 112 – do you have any data regarding how many physiotherapists in Sweden have interest or further training in Womens health, noting PPGP may also be managed by musculoskeletal physiotherapists considering it could come under MSK umbrella**

***Reply:*** *There are approximately 14,000 practising registered physiotherapists in Sweden; however, national data on the number with further training or a specific interest in women's health are not available. As of June 2026, 42 physiotherapists were formally recognised as specialists in obstetrics, gynaecology and urology, with a further 20 in specialist training. Most specialists are located in metropolitan areas. However, specialist certification does not capture all physiotherapists with competence or an interest in women's health. PPGP is also commonly managed within musculoskeletal physiotherapy, and women with PPGP may therefore be treated by physiotherapists without formal specialist qualifications in women's health. We have clarified this in p. 4, lines 112-115.*

**Would be good to include a paragraph that summarises previous qualitative research with women with PPGP and what this has found to then establish the gap your study is filling.**

***Reply:*** *Thank you for this suggestion. We have added a paragraph to the Introduction summarising previous qualitative research on women's experiences of living with PPGP, their experiences of healthcare, and their expectations when seeking physiotherapy. We have also included recent research concerning women's experiences of physiotherapy in primary care. Based on this literature, we have revised the paragraph to more clearly identify the focus of the present study: women's experiences after receiving physiotherapy and whether the care corresponded to their needs and expectations. The study aim has also been clarified accordingly. Please see p. 5 lines 120-139.*

**Method**

**Line 127 – change word "got" for received, eg. All women received oral and written information…**
***Reply:*** *This has been revised, p 5, line 145.*

**Line 131 – can you provide more detail about the timeframe that women received treatment for PPGP. Were they invited to participate after they had complete their physiotherapy sessions or while they were still receiving physiotherapy? This may introduce recall bias if after physiotherapy. How long after were they seen?**

***Reply:*** *The participants were invited four months after delivery, at which time all had completed their physiotherapy for PPGP. We have clarified this timeframe in the Methods, p. 5 lines 148-150, and p. 6, lines153-163. We agree that the retrospective interviews may have introduced some recall bias and have added this to the limitations, p. 20 lines 578-580. However, the purpose of the phenomenological interviews was to explore women's experiences of physiotherapy rather than to establish the factual accuracy of specific treatment details. Participants provided detailed descriptions of their experiences, including their encounters with the physiotherapist and the advice and exercises they had received.*

**Line 144 – Provide more detail regarding how PPGp was verified. What assessments were completed?**

***Reply:*** *We have expanded the Methods (p. 6 lines 166-177) to clarify how PPGP was verified, including the clinical examination, pain location criteria, and requirement for pain reproduction with two or more pelvic pain provocation tests. We have also added a reference to our recently published cross-sectional study, where the assessment procedure is described in greater detail.*

**Line 149 – Provide data regarding how many physiotherapy visits the participants had? Range, average, could add to Table 1 as well**

***Reply:*** *The exact number of physiotherapy visits attended by each participant was not collected and can therefore not be reported. We have instead clarified the treatment structure in the Methods (p. 5 lines 174-177): all participants were scheduled for at least one follow-up visit, with additional sessions offered according to individual needs, treatment response, and time remaining until delivery.*

**How was the sample size determined? With qualitative studies this usually involves saturation of data. Can you please provide information regarding this? (links to SRQR checklist - Sampling strategy)**

***Reply:*** *Data saturation was not used to determine sample size, as saturation is not a methodological requirement in Giorgi's descriptive phenomenological approach. Rather than seeking a point at which no new information emerges, the analysis aims to identify the essential structure of the phenomenon through participants' descriptions of their lived experiences. Recruitment therefore sought to obtain sufficiently rich and varied descriptions to illuminate both commonalities and variations in the phenomenon. P. 18, lines 559-562*

**It looks like you assigned a code to participants for de-identification. Can you please add a line or two about this (links to SRQR checklist – Data processing)**

***Reply:*** *Each participant was assigned a numerical code (1–10) to protect their identity. This information has now been added to the Methods section. p. 7, lines 194-196*

**Did you use an interview guide? How was this developed and piloted? (links to SRQR – Data collection instruments and technologies)**

***Reply:*** *Yes, an interview guide with guiding questions covering different topic areas related to the participants' experiences was used. The interview guide was pilot tested in one interview, which was not included in the analysis. Based on the pilot interview, the opening question was revised. The wording "seeing a physiotherapist" was used instead of the initial wording "receiving physiotherapy," as the latter could imply a narrower focus on treatment received. The revised wording was intended to encourage participants to describe the broader experience of the encounter with the physiotherapist, including the interaction, advice, and treatment, in line with the phenomenological approach. We clarified this on p. 7-8, lines 197-206*

**Did you undertake member checking? If so, please add in details. If not, why not? (links to SRQS – techniques to enhance trustworthiness)**

**Reply:** *Member checking was not performed. In Giorgi's descriptive phenomenological method, the findings represent a phenomenological transformation and synthesis of participants' descriptions rather than a direct representation of individual accounts to be validated by participants. Member checking was therefore not considered an appropriate strategy within the methodological approach used. Instead, rigor was supported through repeated engagement with the transcripts and an iterative, collaborative analytic process involving discussion and revision among the authors. P. 9, lines 238-245.*

**Line 191 – add the following to the sentence, ie. the women's experiences of physiotherapy for PPG, was described as it was .....**
***Reply:*** *This is added in p. 9, lines 234-235*

**Line 195 – the sentence is unfinished, to get a sense of the whole range of topics discussed?, or the whole range of women's narratives? Select one of these or something similar to finish off the sentence.**

**Reply:** *"A sense of the whole" refers to the first step in Giorgi's descriptive phenomenological method, in which the entire description is read before identifying meaning units. To make this clearer, we have revised the sentence to: "The analysis began with repeated readings of each transcript to gain a sense of the whole description before identifying meaning units." P. 9, lines 238-240*

**Results**

**I would suggest moving the quotes to the constituent sections where they would be more illustrative than at the introduction of the theme for all three themes.**

***Reply:*** *The quotes have been moved, and we added some to illustrate the constituents*

**Table 2 – I think the correct grammar would be "Need for proactive and collaborative maternity care rather than "Needs", same for Line 340 and in the Abstract section under results.**
*Reply: Thank you for pointing this out, it has been corrected accordingly.*

**Discussion**

**Another strength is that your themes are similar to other studies that have been conducted elsewhere in the world, eg. refs 23, 24, 26, 30**

***Reply:*** *We agree that the consistency of our findings with those reported in qualitative studies conducted in other settings supports the relevance of the experiences identified beyond the*

*specific study context. We have added this consideration to the Strengths and limitations section, p. 18-19 lines 537-552.*

**Were all your authors women? This may be a limitation worth noting as well if so.**

***Reply:*** *All authors are women and have personal experience of pregnancy, as stated in the Methods. We acknowledge that this shared experience may have influenced our understanding and interpretation of participants' descriptions, while also providing familiarity with the context of pregnancy. In accordance with the descriptive phenomenological approach, we sought to maintain a phenomenological attitude throughout the analysis by reflecting on and restraining our pre-understandings and remaining attentive to the participants' descriptions. We have added this consideration to the Strengths and limitations section, p. 19 lines 582-586.*

**Would also be of benefit to include a few lines about the gap in evidence your research has filled, ie. there are clear links with previous studies here but what is unique about your study? What particular gap has been filled?**

***Reply:*** *We agree that the specific contribution of the present study should be clearer. We have added text to the Discussion (p. 19, lines 545-548) highlighting how our findings extend previous qualitative research. While previous studies have described women's experiences of living with PPGP and their expectations when seeking physiotherapy, our study provides insight into women's experiences after receiving physiotherapy, including how the care received corresponded to their needs and expectations. This has now been more explicitly discussed in relation to previous findings*.

---

## VERSION 2 - REVIEW

---

| | |
|---|---|
| **Reviewer** | **1** |
| **Name** | **Mose, Ayenew** |
| **Affiliation** | **Monash University, Obstetrics and Gynaecology** |
| **Date** | **18-Sep-2026** |
| **COI** | |

---

The authors did great work. Congratulations.
