# Supplementary material for: Women’s experiences of physiotherapy for pregnancy-related pelvic girdle pain in primary care: a qualitative study at four months postpartum
Source: BMJ Open. 2026 Oct 5;16(10):e125092. doi: 10.1136/bmjopen-2026-125092 (PMC13646931; doi:10.1136/bmjopen-2026-125092)
Supplement: Supplementary file 2 [file bmjopen-16-10-s001.docx]

**Interview guide**

This English translation was made only to increase understanding. In the study, all interviews were performed in Swedish.

------------------------------------------------------------------------------------------------------------------------

All participants were asked the same opening question. Depending on how the conversation developed, the topic areas could be addressed in different order and in different ways. The guiding questions for each topic could be used as prompts and when needed to deepen the conversation; the women did not need to specifically answer all questions.

**Opening question**

**“I am interested in hearing about your experience of seeing a physiotherapist for the pelvic girdle pain you had during your most recent pregnancy. Could you tell me about your situation when you decided to seek healthcare for your symptoms?”**

- How did you feel about having to seek healthcare?
- Did you have any expectations before meeting the physiotherapist?
- Was there anything that was particularly important to you when you decided to seek healthcare?

**Topic areas**

**Experience of physiotherapy treatment**

“Could you tell me about the type of advice/treatment you received?”

- How did you experience the treatment/advice/instructions?
- What do you think contributed to you experiencing the treatment in that way?
- Could anything have been done differently? If so, in what way?

**Experience of the encounter with the physiotherapist**

“How did you experience your encounter with the physiotherapist?”

- Were you satisfied with the encounter? Why/why not?
- What about the encounter contributed to that experience?
- Could anything have been done differently? If so, in what way?

**Experience of whether needs/expectations were met**

“Were the needs/expectations you had before the treatment met?”

- In what way were they met? What was not met?
- How did you feel about your needs/expectations being met or not being met?
- Could anything have been done differently? If so, in what way?

**Thoughts on the care of women with pelvic girdle pain in general**

“If you could choose freely, what would you like healthcare and the management of pregnant women with pelvic girdle pain to look like?”

- What is most important to you?

**Summary/closing**

“If I summarize what you have told me…”

- Have I understood you correctly? Is there anything else you would like to add? Something you come to think of regarding treatment of pelvic girdle pain in pregnancy?

**Additional questions/prompts, if needed**

- That was interesting… could you tell me more?
- You mentioned that… could you describe that in more detail?
- What were you thinking/feeling at that time?
- When you said… could you explain what you meant?
